# Aberrant methylation of cell-free circulating DNA in plasma predicts poor outcome in diffuse large B cell lymphoma

**DOI:** 10.1186/s13148-016-0261-y

**Published:** 2016-09-07

**Authors:** Lasse Sommer Kristensen, Jakob Werner Hansen, Søren Sommer Kristensen, Dorte Tholstrup, Laurine Bente Schram Harsløf, Ole Birger Pedersen, Peter De Nully Brown, Kirsten Grønbæk

**Affiliations:** 1 Department of Haematology, Rigshospitalet, Blegdamsvej 9, Copenhagen, Denmark; 2 Department of Clinical Immunology, Næstved Sygehus, Ringstedgade 77A, Næstved, Denmark

**Keywords:** Diffuse large B cell lymphoma, Plasma, Circulating DNA, Liquid biopsy, DNA methylation, Epigenetics, Prognostic markers, Biomarker

## Abstract

**Background:**

The prognostic value of aberrant DNA methylation of cell-free circulating DNA in plasma has not previously been evaluated in diffuse large B cell lymphoma (DLBCL). The aim of this study was to investigate if aberrant promoter DNA methylation can be detected in plasma from DLBCL patients and to evaluate this as a prognostic marker. Furthermore, we wanted to follow possible changes in methylation levels during treatment. Seventy-four patients were enrolled in the study, of which 59 received rituximab and CHOP-like chemotherapy. Plasma samples were collected from all patients at the time of diagnosis and from 14 healthy individuals used as controls. In addition, plasma samples were collected during and after treatment for surviving patients. In total, 158 plasma samples were analyzed for DNA methylation in the promoter regions of *DAPK* (*DAPK1*), *DBC1*, *MIR34A*, and *MIR34B/C* using pyrosequencing.

**Results:**

Aberrant methylation levels at the time of diagnosis were detected in 19, 16, 8, and 10 % of the DLBCL plasma samples for *DAPK1*, *DBC1*, *MIR34A*, and *MIR34B/C*, respectively. *DAPK1* methylation levels were significantly correlated with *DBC1* and *MIR34B/C* methylation levels (*P* < 0.001). For the entire cohort, 5-year overall survival (OS) rates were significantly lower in the groups carrying aberrant *DAPK1* (*P* = 0.004) and *DBC1* (*P* = 0.044) methylation, respectively. *DAPK1* methylation status were significantly correlated with stage (*P* = 0.015), as all patients with aberrant *DAPK1* methylation were stages III and IV. Multivariate analysis identified *DAPK1* as an independent prognostic factor for OS with a hazard ratio of 8.9 (95 % CI 2.7–29.3, *P* < 0.0007). Patients with *DAPK1* methylated cell-free circulating DNA at time of diagnosis, who became long-term survivors, lost the aberrant methylation after treatment initiation. Conversely, patients that maintained or regained aberrant *DAPK1* methylation died soon thereafter.

**Conclusions:**

Aberrant promoter methylation of cell-free circulating DNA can be detected in plasma from DLBCL patients and hold promise as an easily accessible marker for evaluating response to treatment and for prognostication. In particular, aberrant *DAPK1* methylation in plasma was an independent prognostic marker that may also be used to assess treatment response.

**Electronic supplementary material:**

The online version of this article (doi:10.1186/s13148-016-0261-y) contains supplementary material, which is available to authorized users.

## Background

Diffuse large B cell lymphoma (DLBCL) is the most prevalent subtype of B cell non-Hodgkin lymphoma [1]. The prognosis is relatively good for patients responding to the current standard-of-care treatment: rituximab combined with cyclophosphamide, doxorubicin, vincristine, and prednisolone (R-CHOP). However, about one third of the patients, who do not respond or relapse within 5 years after treatment, have a poor prognosis [2, 3], and for DLBCL patients in first complete remission (CR), there is no survival benefit associated with routine imaging [4].

Thus, there is a need for molecular markers that can rapidly and accurately identify patients who will not respond to the treatment or have an increased risk of relapse, as well as markers to assess treatment response, and to detect minimal residual disease (MRD) [5].

Methylation of cytosine residues within the CpG dinucleotide plays important roles in transcriptional regulation during differentiation of normal human cells, but aberrant methylation patterns are also involved in most human cancers [6], including B cell lymphomas [7]. Recent studies have shown that epigenetic modifiers, such as *EZH2*, *MLL2*, and *TET2*, are frequently mutated in DLBCL and associated with altered epigenetic patterns [8–10]. Interestingly, it has also been shown that relapsing patients have more intra-tumor DNA methylation heterogeneity at diagnosis compared to relapse-free patients [11].

Silencing of tumor suppressor genes in cancer often involves methylation of their promotor regions, and the detection of these methylation events in a large range of different tissues shows great promise as diagnostic, prognostic, and predictive markers in various human cancers [12, 13]. Ideally, DNA methylation markers should be detectable in readily accessible body fluids or tissues.

Solid tumors are known to shed cell-free circulating DNA (cfDNA) into the bloodstream, which can be isolated from plasma or serum. Normal cells, including leukocytes, also shed cfDNA into the bloodstream; however, it has been shown that individuals with solid cancers harbor the same mutations and methylation patterns in cfDNA from plasma as in their tumor cells [14].

Tumor-derived cfDNA in serum and plasma has been detected in patients with DLBCL based on next-generation sequencing (NGS) of tumor-specific recombination of the immunoglobulin genes [15–17]. It has also recently been shown that somatic mutations reflecting the genetic changes of primary tumors can be detected in cfDNA from DLBCL patients at time of diagnosis [18, 19]. While these studies prove the usefulness of detecting tumor-derived cfDNA in DLBCL, much more work is clearly needed before standardized tests of cfDNA can be applied in routine clinical settings [5].

In this contribution, we focus on the detection of tumor-derived cfDNA in plasma, as serum is likely to contain DNA derived from leukocytes which lyse during serum processing [20, 21]. Furthermore, we focused on the detection of DNA methylation in the promoter regions of the tumor suppressor genes *DAPK1*, *DBC1*, *MIR34A*, and *MIR34B/C*, which have been shown to be frequently methylated in DLBCL biopsies [22–25]. In addition, methylation of *DAPK1* has been shown to be an independent prognostic factor in DLBCL [22, 26], but none of these markers have been investigated in easily accessible tissues, such as plasma. We hypothesized that aberrant promoter DNA methylation can be detected in plasma from DLBCL patients and have prognostic value. Furthermore, we hypothesized that aberrant promoter DNA methylation in plasma may serve as a marker to assess treatment response.

## Methods

### Patient samples

This retrospective study examined material from 74 DLBCL patients treated at Rigshospitalet, Denmark, who had been diagnosed with DLBCL based on standard histology and immunophenotyping according to the WHO guidelines. None of the patients were under treatment for another malignancy at time of inclusion. Peripheral blood (PB) plasma was collected from all patients at the time of diagnosis and 14 days after the fourth and last treatment cycle, respectively, and 3 months after end of treatment from surviving patients. In addition, PB plasma samples were collected from 14 healthy blood donors from the Danish Blood Donor Study [27]. The patients were diagnosed from 2003 to 2007 and at least 5 years of clinical follow-up were available for all patients except three.

### DNA extraction and sodium bisulfite conversion

DNA extractions from plasma were performed with the ROCHE MagNa Pure, using the MagNA Pure LC Total Nucleic Acid Isolation kit (Roche Diagnostics, Mannheim, Germany) for all plasma samples from the normal controls, and the patient samples from time of diagnosis and end of treatment. The QIAsymphony Circulating NA Kit (48), cus, G (QIAGEN, Hilden, Germany) was used for the samples collected during treatment. DNA concentrations were measured using the Qubit flourometer (ThermoFisher Scientific, Waltham, MA, USA). Between 10 and 100 ng, DNA were converted with the EZ DNA Methylation kit (Zymo Research, Irvine, CA, USA) according to the manufactures’ instructions.

### DNA methylation detection using pyrosequencing

Traditional methylation-independent PCR pyrosequencing assays [28] were designed to target the promoter regions of *DAPK1*, *DBC1*, *MIR34A*, and *MIR34B/C*. The PCR and sequencing primers were designed using the PyroMark Assay Design 2.0 software (QIAGEN). The primer sequences and information about the assays can be found in (Additional file 1: Table S1). PCR cycling was performed on the Gene PCR System 9700 (Applied Biosystems, Foster City, CA, USA). The cycling protocol started with 1 cycle of 95 °C for 15 min, followed by 45 cycles of 95 °C for 20 s, 58 °C (60 °C for the *DAPK1* assay) for 20 s, 72 °C for 20 s, and 1 cycle of 72 °C for 10 min. For the reaction mixtures, the PyroMark PCR Master Mix (QIAGEN) was used at a final concentration at 1×, resulting in a final MgCl2 concentration of 1.5 mM. Final primer concentrations were 200 nM and 1 μL bisulfite converted DNA was used as template. Samples were sequenced on the PyroMark Q24 (QIAGEN) using the PyroMark Gold Q24 reagents (QIAGEN), according to the manufactures’ instructions. Methylated DNA (Chemicon, Millipore, Billerica, MA), unmethylated DNA (QIAGEN), and a no template control (NTC) were included in all experiments. Aberrant methylation was defined as a methylation level above the mean methylation level plus two standard deviations of the control group. The cutoffs were 5.5, 20.9, 4.2, and 7.8 % for *DAPK1*, *DBC1*, *MIR34A*, and *MIR34B/C*, respectively.

### Statistical analyses

Statistical analyses were performed in SPSS 19.0 for Windows (SPSS Inc.) and in Prism 6 (GraphPad software, San Diego, CA, USA). Goodness-of-fit linear regression was used to evaluate possible relations between methylation levels and DNA concentrations as well as *DAPK1* methylation levels and methylation levels of the other markers by employing an F test to evaluate if the slopes were significantly different from zero. Correlations between 5-year overall survival (OS) rates and methylation status as well as clinical characteristics were estimated using the Kaplan-Meier method with the use of a log-rank test. Hazard ratios (HR) and 95 % confidence intervals (CI) were calculated using a univariate Cox proportional hazard model. The clinical characteristics and treatment outcomes were compared according to *DAPK1* methylation status using Students *T* tests, Pearson’s chi-square tests, or Fisher’s exact tests when expected values were below five. For assessment of independent predictors of OS, a multivariate Cox regression hazard model was applied. Any differences were considered to be statistically significant when the *P* value was <0.05.

## Results

### cfDNA concentrations in the plasma samples

**Fig. 1:**
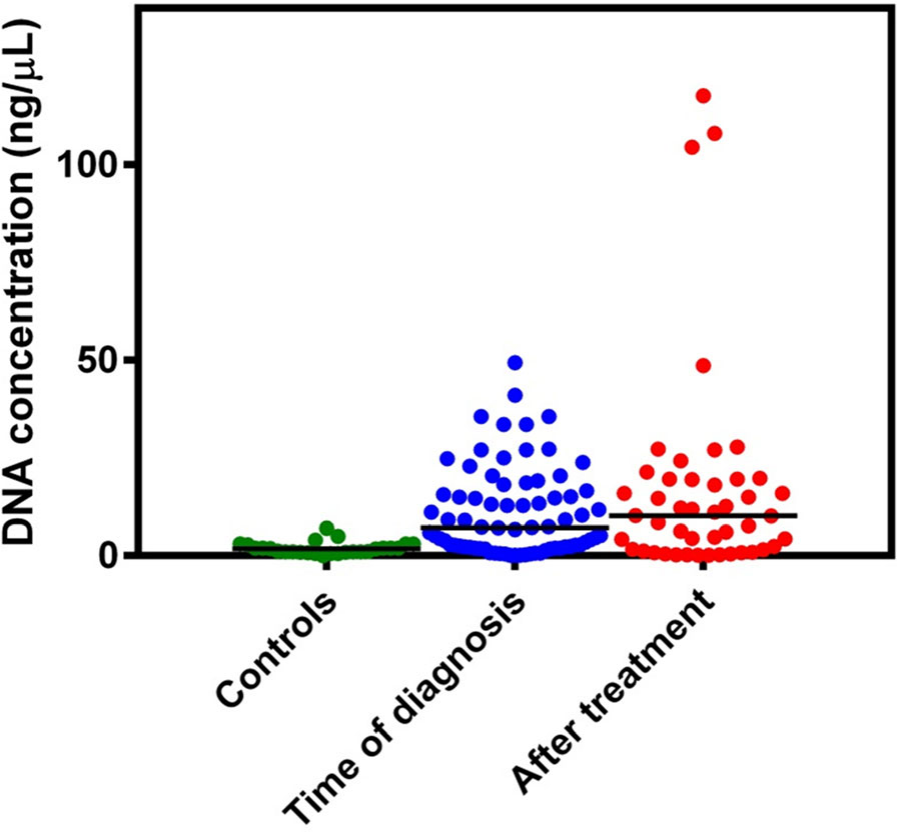
DNA concentrations in the control plasma samples and the plasma samples taken at time of DLBCL diagnosis and following treatment

The cfDNA concentrations were significantly higher for the samples collected at time of diagnosis compared to the normal control samples (*P* < 0.0001), while there was no difference between the samples from time of diagnosis and the samples collected after treatment (Fig. 1).

### Detection of aberrant methylation in the plasma samples

**Fig. 2:**
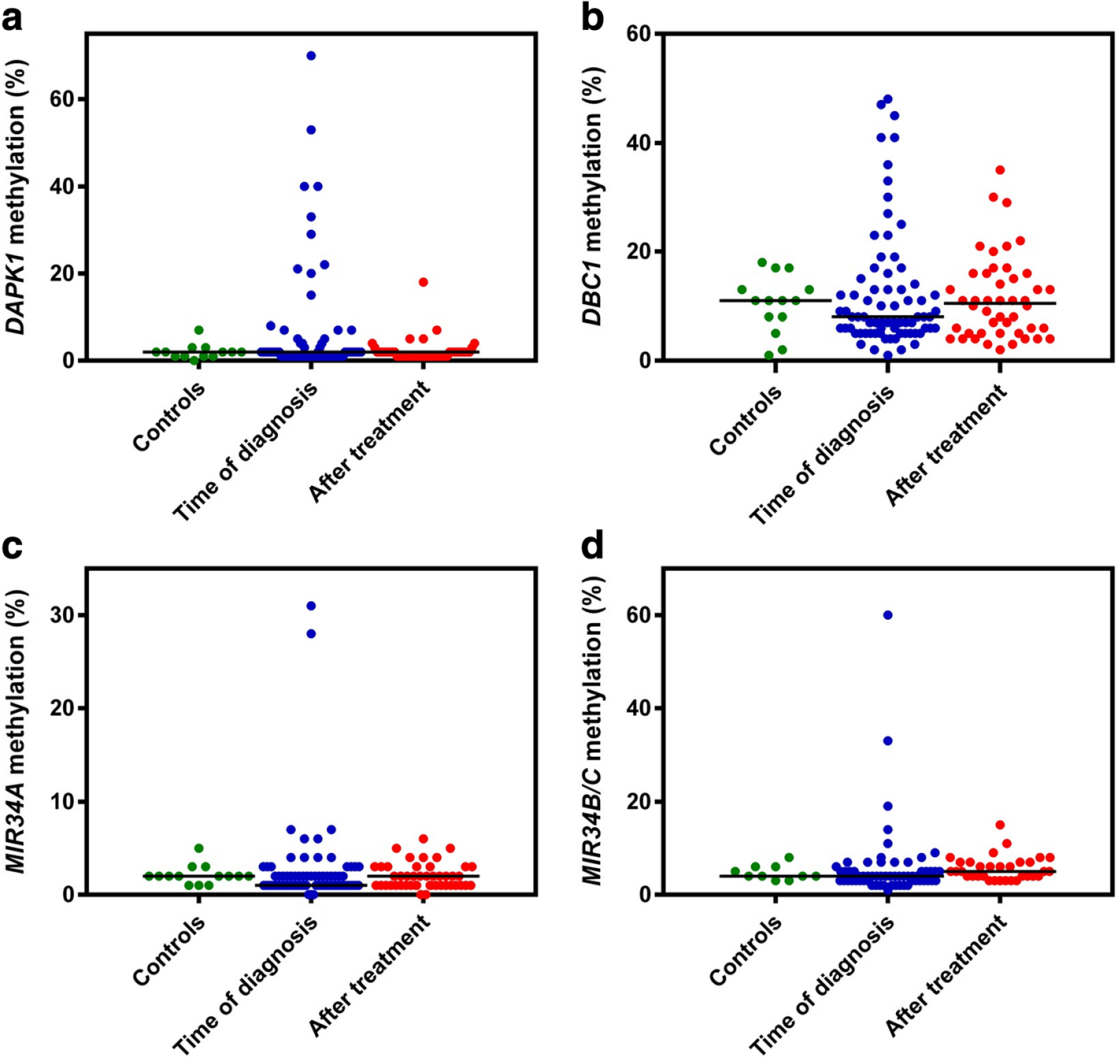
Methylation levels in the plasma samples. The *solid lines* represent medians. **a** *DAPK1* methylation levels. **b** *DBC1* methylation levels. **c** *MIR34A* methylation levels. **d** *MIR34B/C* methylation levels

The methylation analyses were successful for all samples for all markers except *MIR34B/C*, which failed for 12 and 13 of the diagnostics samples and samples collected after end of treatment, respectively. For *DAPK1* aberrantly methylated DNA in plasma was detected in 14 (19 %) of the patients at the time of diagnosis, while only 2 (4 %) were methylated after end of treatment. For *DBC1*, *MIR34A*, and *MIR34B/C*, aberrantly methylated DNA were detected in 12 (16 %), 6 (8 %), and 6 (10 %) of the patients at the time of diagnosis, respectively, while 6 (13 %), 4 (9 %), and 5 (15 %) were methylated after end of treatment, respectively (Fig. 2). *DAPK1* methylation levels were significantly correlated with *DBC1* and *MIR34B/C* (*P* < 0.001 for both), but not with *MIR34A* methylation levels (Additional file 2: Figure S1). None of the markers were correlated with the DNA concentration in the samples (Additional file 3: Figure S2).

### Survival analyses according to methylation status and DNA concentrations

The prognostic value of methylated cfDNA levels at time of diagnosis was evaluated by analyzing OS rates according to each of the four markers for all patients with clinical data available (*n* = 71) and in separate analyses including only patients receiving rituximab (*n* = 59).

**Fig. 3:**
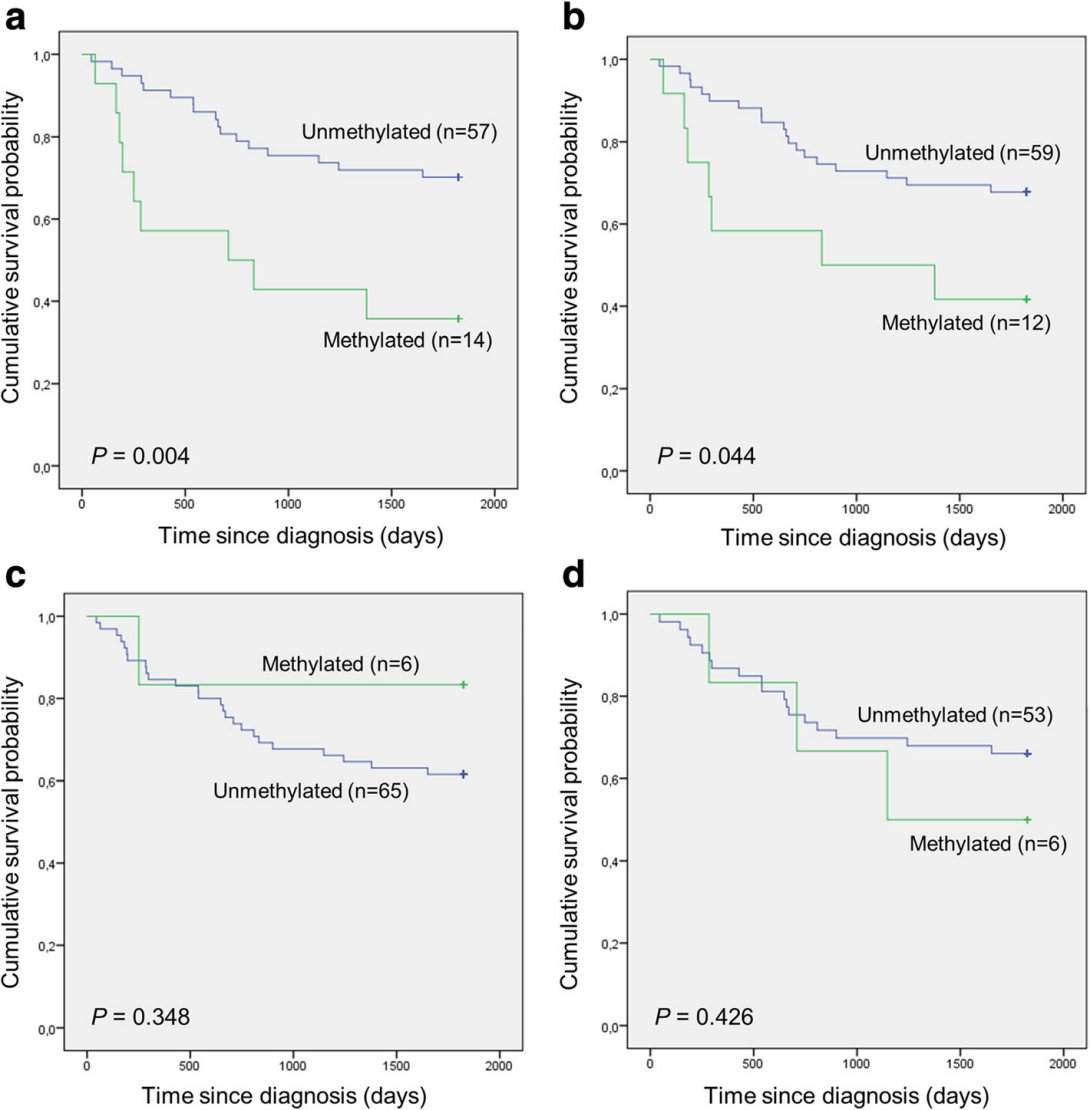
Overall survival of the entire cohort according to methylation status. **a** *DAPK1*. **b** *DBC1*. **c** *MIR34A*. **d** *MIR34B/C*

When analyzing all patients, the 5-year OS rates were 35.7 and 70.0 % in the *DAPK1* methylated group and unmethylated group, respectively. The hazard ratio for death in the methylated group was 3.08 (95 % CI, 1.37 to 6.93; *P* = 0.004). The 5-year OS rates were 41.7 and 67.8 % in the *DBC1* methylated and unmethylated group, respectively. The hazard ratio for death in the methylated group was 2.38 (95 % CI, 1.00 to 5.66; *P* = 0.044). The 5-year OS rates were 83.3 and 60.9 % in the *MIR34A* methylated and unmethylated group, respectively. The hazard ratio for death in the methylated group was 0.40 (95 % CI, 0.05 to 2.93; *P* = 0.348). The 5-year OS rates were 50.0 and 67.9 % in the *MIR34B/C* methylated and unmethylated group, respectively. The hazard ratio for death in the methylated group was 1.64 (95 % CI, 0.48 to 5.60; *P* = 0.426) (Fig. 3).

Thirty-eight of the patient samples had elevated DNA concentrations compared to the normal controls (Fig. 1). When analyzing all patients, the 5-year OS rates were 63.2 and 63.6 % in the group with elevated DNA concentrations and the group with normal DNA concentrations, respectively. The hazard ratio for death in the group with elevated DNA concentrations was 0.98 (95 % CI, 0.45 to 2.12; *P* = 0.963) (Additional file 4: Figure S3).

The 5-year OS rates for patients receiving rituximab were 41.7 and 70.2 % in the *DAPK1* methylated and unmethylated group, respectively. The hazard ratio for death in the methylated group was 2.56 (95 % CI, 1.03 to 6.35; *P* = 0.036), and the 5-year OS rates were 50.0 and 67.3 % in the *DBC1* methylated and unmethylated group, respectively. The hazard ratio for death in the methylated group was 1.80 (95 % CI, 1.03 to 4.92; *P* = 0.245) (Additional file 5: Figure S4).

### *DAPK1* methylation status according to patient characteristics

The clinical characteristics of the DLBCL patients as a function of *DAPK1* methylation status are shown in Table 1. No significant differences in patient demographics or disease characteristics according to *DAPK1* methylation status were observed, with the exception of stage, as all patients with aberrant *DAPK1* methylation had stage III or IV cancer. This was expected as it has been shown in solid tumors that patients with an advanced stage are more likely to have detectable tumor-derived cfDNA in plasma as compared to patients with localized disease [29].

**Table 1:** Clinical characteristics of the DLBCL patients according to *DAPK1* methylation status

	Total (*n* = 71)	Unmethylated (*n* = 57)	Methylated (*n* = 14)	*P* value
Sex				0.136
Men	38	33	5	
Women	33	24	9	
Extranodal involvement				0.098
Yes	53	40	13	
No	18	17	1	
Stage				0.015
I–II	18	18	0	
III–IV	53	39	14	
Elevated LDH^b^				1.000
Yes	51	40	11	
No	17	14	3	
B symptoms^b^				1.000
Yes	20	16	4	
No	49	39	10	
IPI score^b^				0.404
0–2	26	22	4	
3–5	42	32	10	
Performance score^a^				1.000
0–2	62	50	12	
3–4	9	7	2	
Response^b^				0.367
CR	51	43	8	
PD/PR	10	7	3	
Mors	4	2	2	
Age at diagnosis (grouped)				0.959
<60 years	30	24	6	
≥60 years	41	33	8	
Age at diagnosis				0.653
Age, mean (range)	60 years (23–85)	60 years (23–85)	59 years (35–72)	
Rituximab^b^				1.000
Yes	59	47	12	
No	11	9	2	

*LDH* lactate dehydrogenase, *IPI* international prognostic index, *CR* complete response, *PD* progressive disease, *PR* partial response

^a^Eastern Cooperative Oncology Group

^b^Data was not available for all patients

### Multivariate analysis of survival according to *DAPK1* methylation status

For most baseline risk factors, including male sex, age above 60 years, extranodal involvement, poor performance score, and high IPI, a trend towards a poor OS was observed as expected. However, this was not statistically significant for any of them, possible due to the limited sample size (Table 2). Therefore, the fact that aberrant *DAPK1* methylation was a highly significant predictor of overall survival in this cohort of limited size emphasize its high potential as a prognostic marker in DLBCL. To further substantiate our findings, we performed multivariate analyses of survival according to *DAPK1* methylation status. All baseline risk factors (gender, age, LDH, extranodal involvement, performance score, stadium, B symptoms, and treatment with rituximab) were included in the Cox proportional hazard model. The model identified methylation of *DAPK1* as an independent prognostic factor for OS. The HR for *DAPK1* methylation was 8.9 (95 % CI 2.7–29.3, *P* < 0.0007). None of the other baseline risk factors were significant with the exception of male sex and poor performance score (Table 3).

**Table 2:** Impact of clinicopathological parameters on OS in DLBCL

	Total (*n* = 71)	Number of events	*P* value
Sex			0.219
Men Women	38 33	16 10	
Extranodal involvement			0.068
Yes No	53 18	23 3	
Stage			0.520
I–II III–IV	18 53	8 18	
Elevated LDH^b^			0.210
Yes No	51 17	16 8	
B symptoms^b^			0.149
Yes No	20 49	10 15	
IPI score^b^			0.431
0–2 3–5	26 42	8 16	
Performance score^a^			0.114
0–2 3–4	62 9	21 5	
Response^b^			0.061
CR PD/PR	51 10	14 5	
Age at diagnosis			0.059
<60 years ≥60 years	30 41	7 19	
Rituximab^b^			0.782
Yes No	59 11	21 4	

*LDH* lactate dehydrogenase, *IPI* international prognostic index, *CR* complete response, *PD* progressive disease, *PR* partial response

^a^Eastern Cooperative Oncology Group

^b^Data was not available for all patients

**Table 3:** Multivariate Cox regression analyses for baseline risk factors in DLBCL

Baseline risk factor	Hazard ratio	95 % hazard ratio confidence limits		*P* value
		Lower	Upper	
Age above 60 years	2.85	0.97	8.41	0.058
Male sex	4.10	1.41	11.92	0.010
Poor performance	5.36	1.56	18.41	0.008
Stage (III–IV)	0.44	0.13	1.42	0.169
Extranodal involvement	1.32	0.34	5.07	0.687
B symptoms	1.80	0.68	4.80	0.238
Not treated with rituximab	0.55	0.15	2.10	0.383
Elevated LDH	0.73	0.26	2.06	0.553
*DAPK1* methylation	8.93	2.72	29.31	0.0007

### Changes in methylation levels during treatment

**Fig. 4:**
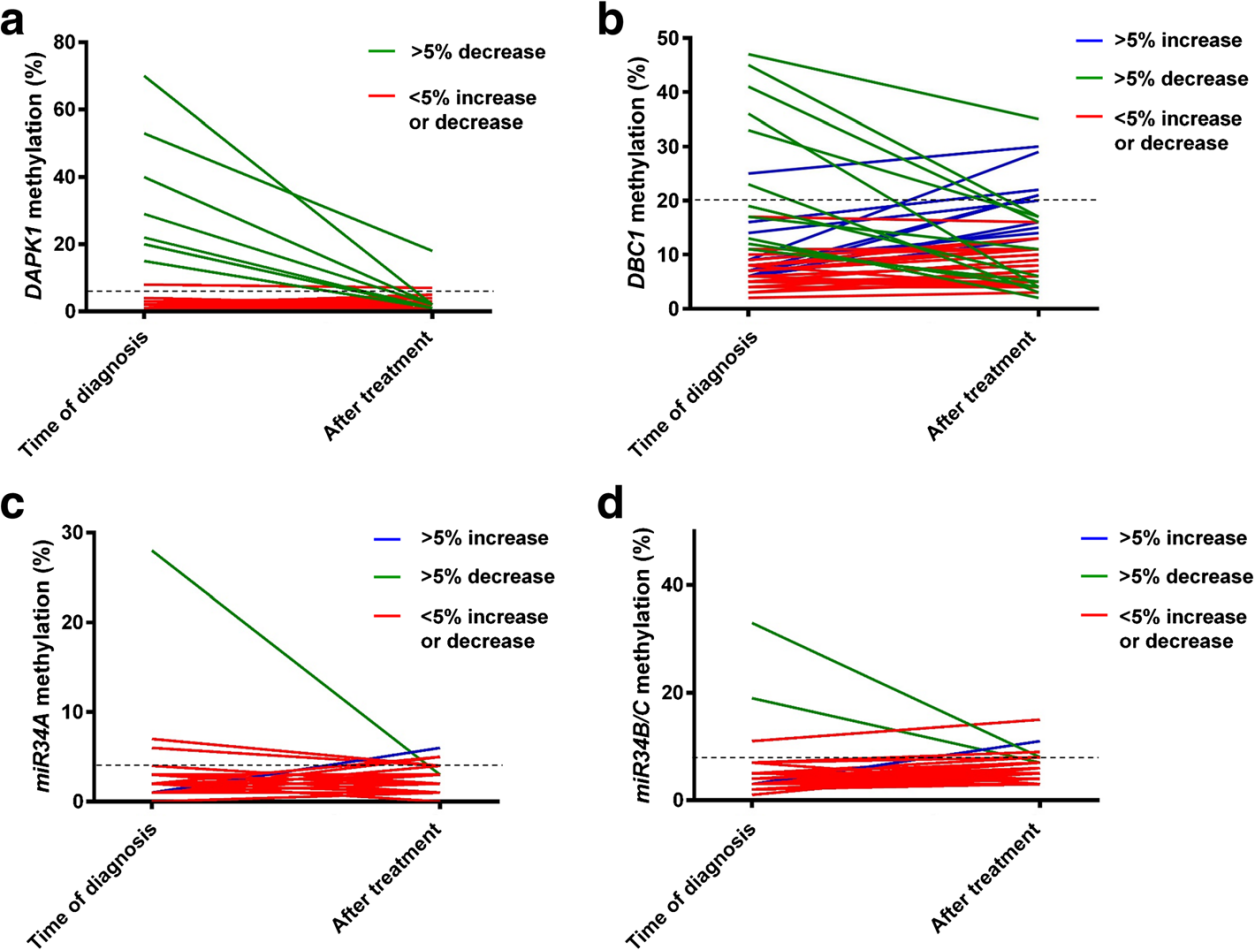
Methylation levels before and after treatment for surviving patients. Each *solid line* represents a sample pair. The *dashed lines* represent the individual cutoffs for aberrant methylation. **a** *DAPK1*. **b** *DBC1*. **c** *MIR34A*. **d** *MIR34B/C*

Methylation levels for each of the markers were compared between the diagnostic sample and the sample collected 3 months after the end of treatment for each of the individual surviving patients. All patients with aberrant *DAPK1* methylation at the time of diagnosis who were alive after the end of treatment showed a decrease in methylation levels, and all except two patients had methylation levels within the normal range after treatment (Fig. 4a). For *DBC1*, 11 of the patients showed a greater than 5 % decrease in methylation levels, while 10 of the patients showed a greater than 5 % increase. Altogether, aberrant methylation was detected in six of the patients after treatment (Fig. 4b). For *MIR34A*, one patient showed a greater than 5 % decrease in the methylation level, and one patient showed a greater than 5 % increase. Altogether, aberrant methylation was detected in four of the patients after treatment (Fig. 4c). For *MIR34B/*C, two patients showed a greater than 5 % decrease in methylation levels, and one patient showed a greater than 5 % increase. Altogether, aberrant methylation was detected in five of the patients after treatment (Fig. 4d).

**Fig. 5:**
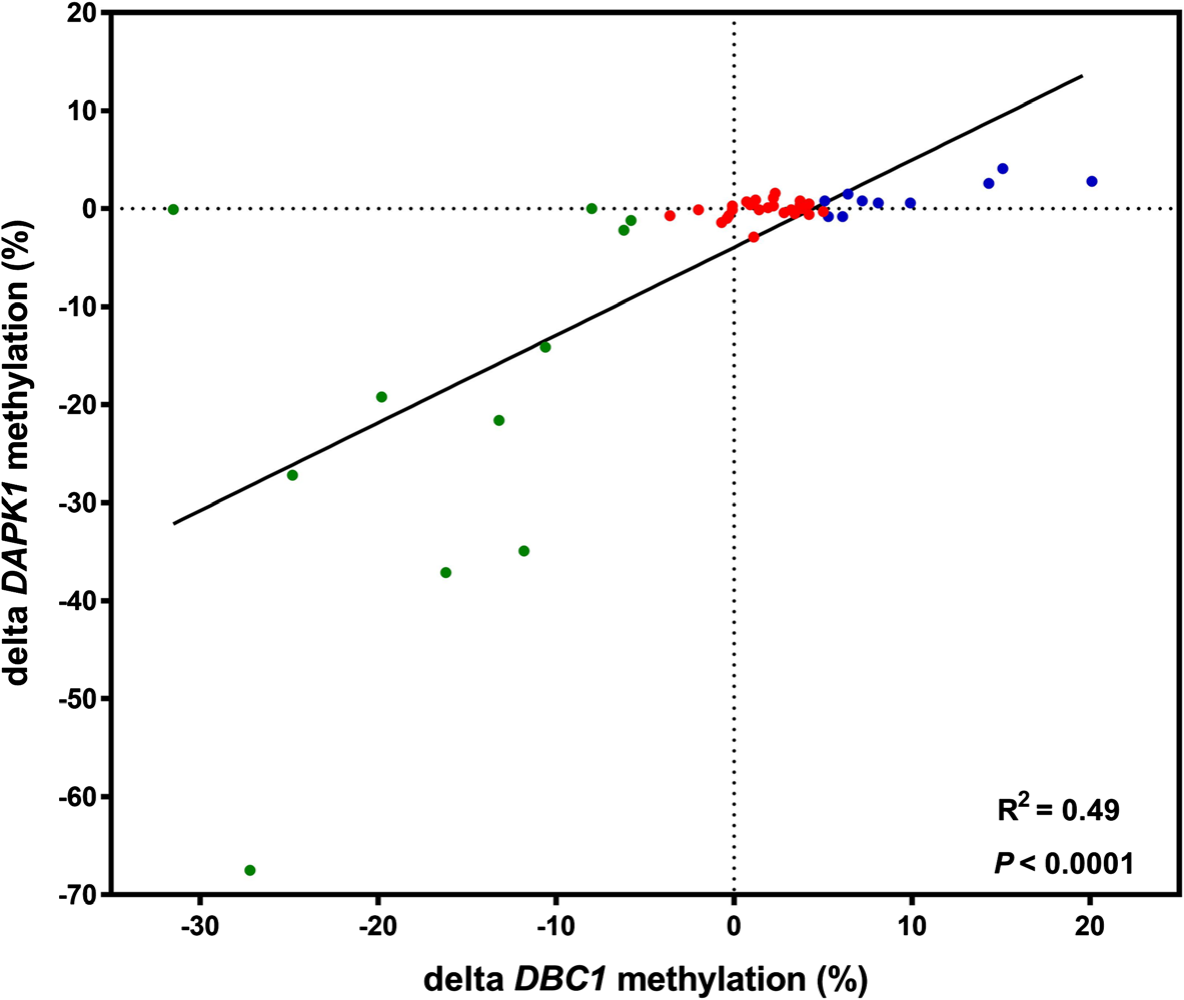
Correlation between changes in *DAPK1* and *DBC1* methylation levels during treatment

None of the patients had a decrease in *DAPK1* methylation and an increase in *DBC1* methylation and vice versa (Fig. 5), indicating that the aberrant methylation is tumor-specific.

**Fig. 6:**
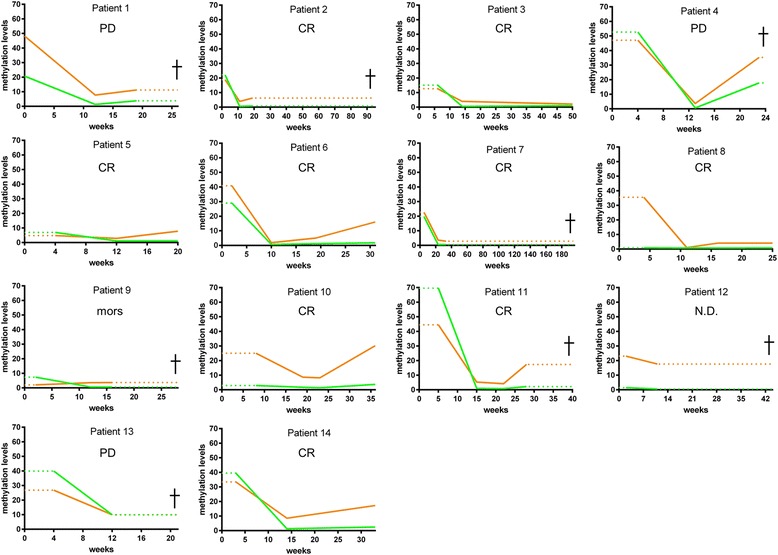
Methylation levels during treatment. Each plot shows the *DAPK1* (*green lines*) and *DBC1* (*orange lines*) methylation data acquired during treatment for an individual patient. Time of diagnosis is at week 0 and the treatment was initiated at the same time or 1–2 weeks after the first methylation measurement (start of *solid lines*). The last methylation measurement is indicated by the end of the *solid lines*. Time of death is indicated by a *cross*. Patients without a cross survived at least 5 years following their diagnosis. Patient 1 progressed to DLBCL from a follicular lymphoma. Response evaluations are displayed for each patient. *CR* complete response, *PD* progressive disease, *N.D.* no data

For 14 of the patients with a *DAPK1* and/or *DBC1* methylated sample at the time of diagnosis, one or two samples collected during treatment were available. These additional samples were tested for *DAPK1* and *DBC1* methylation (Fig. 6). For all of these patients, except patient 13, the *DAPK1* methylation levels decreased to a level within the normal range after the treatment was initiated. Patient 13 relapsed and died 181 days after diagnosis. Six patients survived at least 5 years following diagnosis, and all of these retained a *DAPK1* methylation level within the normal range for all samples collected during and after treatment. One of the patients (patient 4) died less than a week after the *DAPK1* methylation level increased to an aberrant level, and two patients (patients 1 and 11) died 9 and 12 weeks, respectively, after a slight increase in *DAPK1* methylation levels was measured. Two of the patients (patients 7 and 12) survived for more than 30 weeks following their last *DAPK1* methylation measurements, which were within the normal range. The last patient (patient 9) died from the disease 11 weeks after the final *DAPK1* methylation measurement, which was within the normal range. For several of the surviving patients *DBC1* methylation levels increased more than *DAPK1* methylation levels, and one patient (patient 10) increased to an aberrant level, but survived more than 5 years following diagnosis.

Altogether, these analyses demonstrate that *DAPK1* methylation in cfDNA from plasma has a potential as a marker to assess treatment response in DLBCL.

## Discussion

The possibility to assess tumor-specific molecular characteristics simply by analyzing peripheral blood, without the need for an invasive tumor biopsy, has been an objective in molecular diagnostics for long. A breakthrough in this area came with the discovery of tumor-specific cfDNA and circulating tumor cells in plasma and serum, which led to the concept known as “liquid biopsy” [30].

Different strategies have been employed for the detection of tumor-specific cfDNA in DLBCL [16, 17, 19, 31]. Frickhofen et al. detected rearrangements of the IgH locus in plasma and serum as a marker of tumor-specific cfDNA and showed that the presence of tumor-specific DNA may be used for monitoring response to treatment in B cell malignancies [17]. However, patients with a negative test result later relapsed, and the specificity of this approach as a response marker is not clear. He et al. later refined this approach by using NGS followed by PCR to detect specific rearranged IgH DNA fragments in plasma [16], but the usefulness of this approach as a prognostic marker or as a marker of MRD following therapy was not evaluated. In a preliminary study, Hosny et al. detected *TP53* mutations in plasma using direct Sanger sequencing and a PCR-restriction digestion analysis, but no comparisons to clinical outcome were performed [19]. Bohers et al. recently detected tumor-specific cfDNA in plasma by sequencing a panel of 34 genes recurrently mutated in lymphoma using Ion Torrent (Life Technologies) NGS. Their study showed that the mean variant allele frequency can be expected to be lower in cfDNA compared to primary tumor biopsies, and not all mutations present in the tumor samples may be detected in plasma samples [18]. Finally, Jones et al. detected circulating Epstein-Barr virus (EBV) DNA in plasma and serum as a marker of tumor-specific cfDNA, and showed that the presence of tumor-specific DNA correlated with clinical/radiological response. However, this approach is only applicable to EBV-associated lymphoma [31], and it is unclear if the presence of EBV DNA in plasma and/or serum at the time of diagnosis has prognostic value.

We adopted a different approach for the detection of tumor specific cfDNA in DLBCL, namely the detection of aberrant DNA methylation by pyrosequencing. This method is currently in use in clinical settings for the detection of O^6^-methylguanine-DNA methyltransferase (*MGMT*) methylation, which is used as a prognostic and predictive biomarker in glioblastoma treated with alkylating agents [32–34].

As a proof of principle, we selected four tumor suppressor genes, *DAPK1*, *DBC1*, *MIR34A*, and *MIR34B/C*, which have been shown to be frequently methylated in DLBCL [22–25], and investigated if aberrant methylation levels of these markers could be detected in plasma from patients with DLBCL. Aberrant methylation levels were detected for all markers. However, the frequencies of patients with aberrant methylation were lower than what have previously been observed in DLBCL biopsies [22, 24, 25]. This was expected as cfDNA in plasma contains a larger background of DNA from normal cells in most cases as compared to tumor biopsies. Furthermore, it has also been shown for other malignancies that patients harboring solid tumors with advanced disease are more likely to have detectable tumor-derived cfDNA in plasma as compared to patients with localized disease [29]. In line with this, we observed that the presence of aberrant *DAPK1* methylation only occurred for patients with stage III and IV disease.

cfDNA concentrations were elevated at presentation, however, a high DNA concentration did not confer a poor prognosis, and none of the methylation markers we studied were correlated with DNA concentration. This was expected as it has previously been shown that tumor-derived cfDNA, but not non-specific cfDNA, reflects therapeutic response [31], and it has been shown that samples from patients with pediatric anaplastic large cell lymphoma with a high percentage of tumor-derived cfDNA tend to have low concentrations of cfDNA [35]. On the other hand, quantification of cfDNA based on qPCR for the *β-globin* gene, has been shown to correlate with disease characteristics, such as presence of B symptoms, elevated LDH, and advanced stage disease as well as a poor prognosis [36].

While the detection of tumor-derived cfDNA has potential as a prognostic marker, we hypothesized that the detection of markers in cfDNA, which have prognostic value when detected in primary tumor biopsies, may strengthen that potential. Methylation of *DAPK1* in tumor biopsies has proven to be an independent prognostic marker in DLBCL [22, 26] and is also methylated in a large proportion of the patients. Indeed, we found methylation of the *DAPK1* gene to be a strong predictor of OS and it remained statistically significant when correcting for gender, age, LDH, extranodal involvement, performance score, stage, B symptoms, and treatment with rituximab. Compared to *DAPK1* methylation in DLBCL biopsies, a much smaller proportion of the patients have aberrant methylation in cfDNA from plasma, possibly because only the more advanced tumors shed DNA into the circulation. This is very important as it is of exceptional clinical importance to be able to identify a relatively small group of patients who are not responding to current treatment modalities, rather than to identify a small group of patients doing remarkably well, as is the case for patients with an unmethylated DLBCL biopsy [22]. Another important finding was that patients with *DAPK1* methylated cfDNA at time of diagnosis, who became long-term survivors, lost the aberrant methylation after treatment initiation. Conversely, patients that maintained or regained aberrant *DAPK1* methylation died soon thereafter. Thus, based on these data, we suggest that the small group of patients with aberrant *DAPK1* methylation in plasma at the time of diagnosis should be monitored for *DAPK1* methylation during treatment and could potentially be selected for relapse treatment if the methylation level increases. A number of new agents have shown promising results for the treatment of relapsed/refractory DLBCL used as single-agent or in combination with rituximab-based chemotherapy [37].

At this time, it is unknown if *DAPK1* methylation has functional importance for the resistant/relapsing clones. If this is the case, it is tempting to speculate whether DNA methyltransferase inhibitors should be added to the salvage therapy in these patients as azacitidine plus standard chemoimmunotherapy in high-risk patients with newly diagnosed DLBCL is well tolerated and yield a high rate of complete remission [38].

Finally, it may be possible in the future to reconstitute the expression of DAPK1 in patients lacking this important tumor suppressor as delivery of its constitutive kinase domain via a CD22-specific immunoligand has shown remarkable in vitro efficacy and selectivity in preclinical testing [39].

## Conclusions

Aberrant methylation of cfDNA can be detected in plasma from DLBCL patients and methylation of *DAPK1* has a strong potential as an independent prognostic marker and may also be used to assess treatment response. However, these results need to be confirmed in additional independent and larger cohorts of DLBCL patients before testing for *DAPK1* methylation of cfDNA should be incorporated into routine clinical practice. Nevertheless, this study highlights the potential of liquid biopsies in DLBCL as a prognostic tool and for monitoring patients during and following treatment.

## Additional files

Additional file 1: Table S1. Details of the pyrosequencing assays. (TIF 14 kb) Additional file 2: Figure S1.
*DAPKI* methylation levels according to methylation levels of the other markers. (a) *DBCI*. (b) *MIR34A*. (c) *MIR34B/C.* (TIF 990 kb) Additional file 3: Figure S2. Methylation levels according to DNA concentrations. (a) *DAPKI*. (b) *DBCI* (c) *MIR34A*. (d) *MIR34B/C.* (TIF 1 mb) Additional file 4: Figure S3. Overall survival according to DNA concentrations. (TIF 593 kb) Additional file 5: Figure S4. Overall survival of the rituximab treated patients according to methylation status. (a) *DAPKI.* (b) *DBCI*. (TIF 754 kb)
