# Supplementary material for: Aberrant methylation of cell-free circulating DNA in plasma predicts poor outcome in diffuse large B cell lymphoma
Source: Clin Epigenetics. 2016 Sep 7;8(1):95. doi: 10.1186/s13148-016-0261-y (PMC5015248; doi:10.1186/s13148-016-0261-y)
Supplement: Additional file 1: Table S1. — Details of the pyrosequencing assays. (TIF 14 kb) [file 13148_2016_261_MOESM1_ESM.docx]

**Table S1. Details of the pyrosequencing assays**

| **Name of assay** | **Primer sequences (5’ →3’)** | **Amplicon size** |
| --- | --- | --- |
| *DAPK1* | Forward: GGGTAGGGTGAGAATAGGT  Reverse: Biotin-CCCCAAAACCACATTCCT  Sequencing primer: GGTGAGAATAGGTGG | 180 bp |
| *DBC1* | Forward: GTGGGAATTTGGGAGAGTTTT  Reverse: Biotin-CAAACTACTAAAAACCAAATACCCC  Sequencing primer: AATTTGGGAGAGTTTTG | 91 bp |
| *MIR34A* | Forward: GGTTTGGGGATAGTTTAGTTTT  Reverse: Biotin-CCTTCCTACTCCTACCACCAAACC  Sequencing primer: GGATTTAGGGTTGGAGAGA | 83 bp |
| *MIR34B/C* | Forward: GTAGTTTTAGAGAGAAGAAGTTTGAGAAG  Reverse: : Biotin-CTTCTATAACTCCTAAAAAAAACTAAC  Sequencing primer: GAGAAGATGTTTGAGAAG | 148 bp |
