# Supplementary figures and images for: Aberrant methylation of cell-free circulating DNA in plasma predicts poor outcome in diffuse large B cell lymphoma
Source: Clin Epigenetics. 2016 Sep 7;8(1):95. doi: 10.1186/s13148-016-0261-y (PMC5015248; doi:10.1186/s13148-016-0261-y)

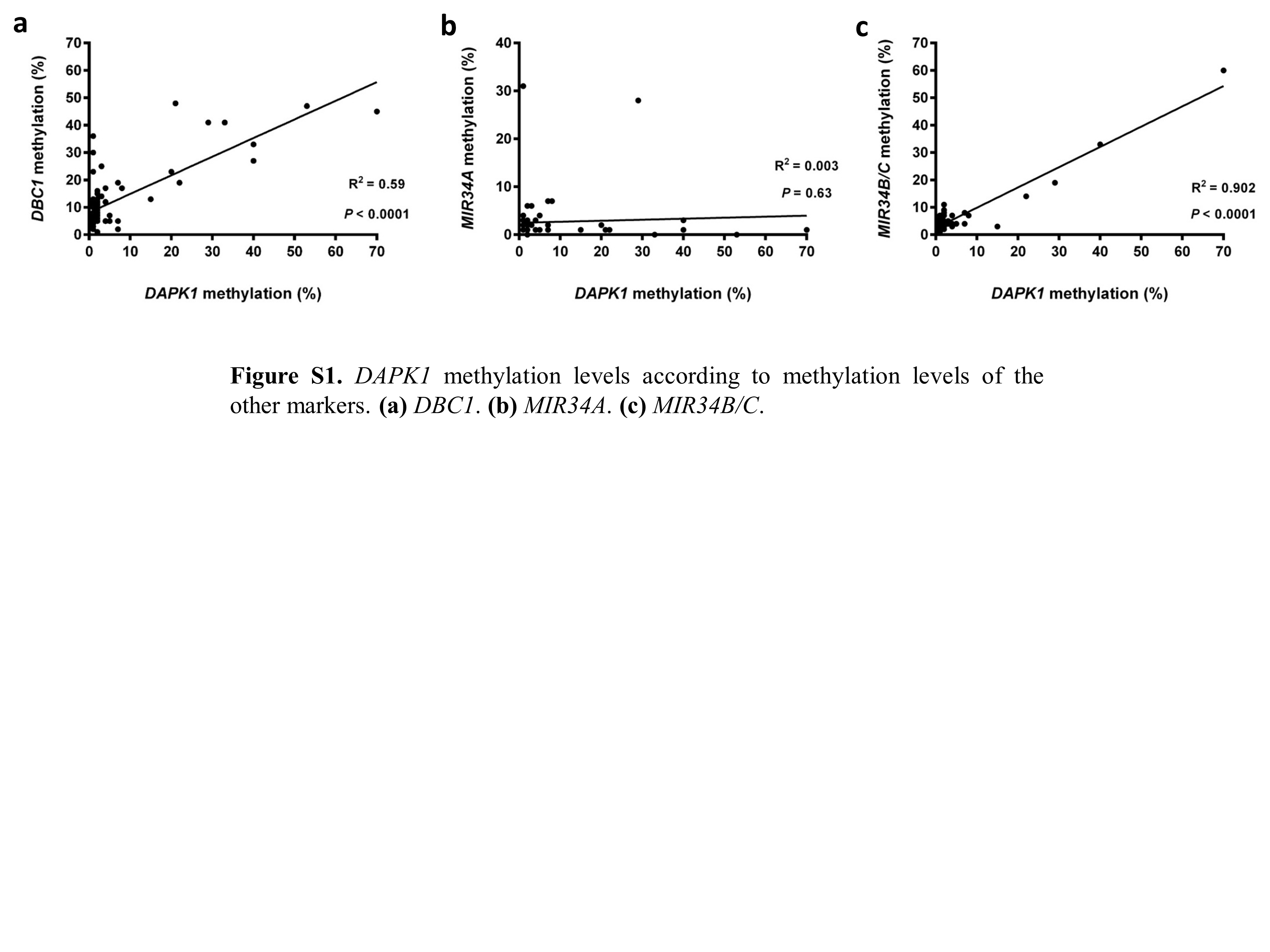

Supplement: Additional file 2: Figure S1. — DAPKI methylation levels according to methylation levels of the other markers. (a) DBCI. (b) MIR34A. (c) MIR34B/C. (TIF 990 kb) [file 13148_2016_261_MOESM2_ESM.tif]

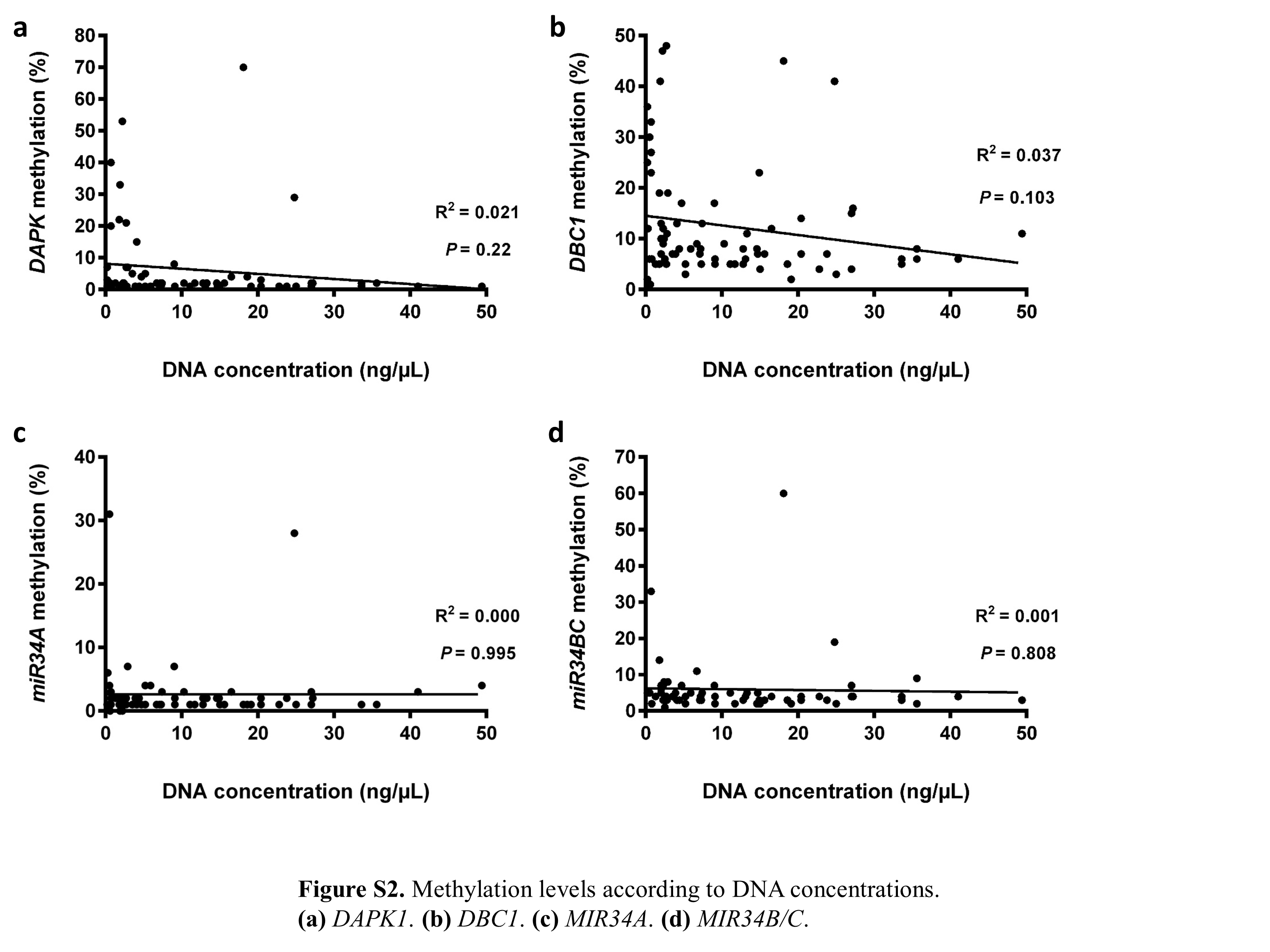

Supplement: Additional file 3: Figure S2. — Methylation levels according to DNA concentrations. (a) DAPKI. (b) DBCI (c) MIR34A. (d) MIR34B/C. (TIF 1 mb) [file 13148_2016_261_MOESM3_ESM.tif]

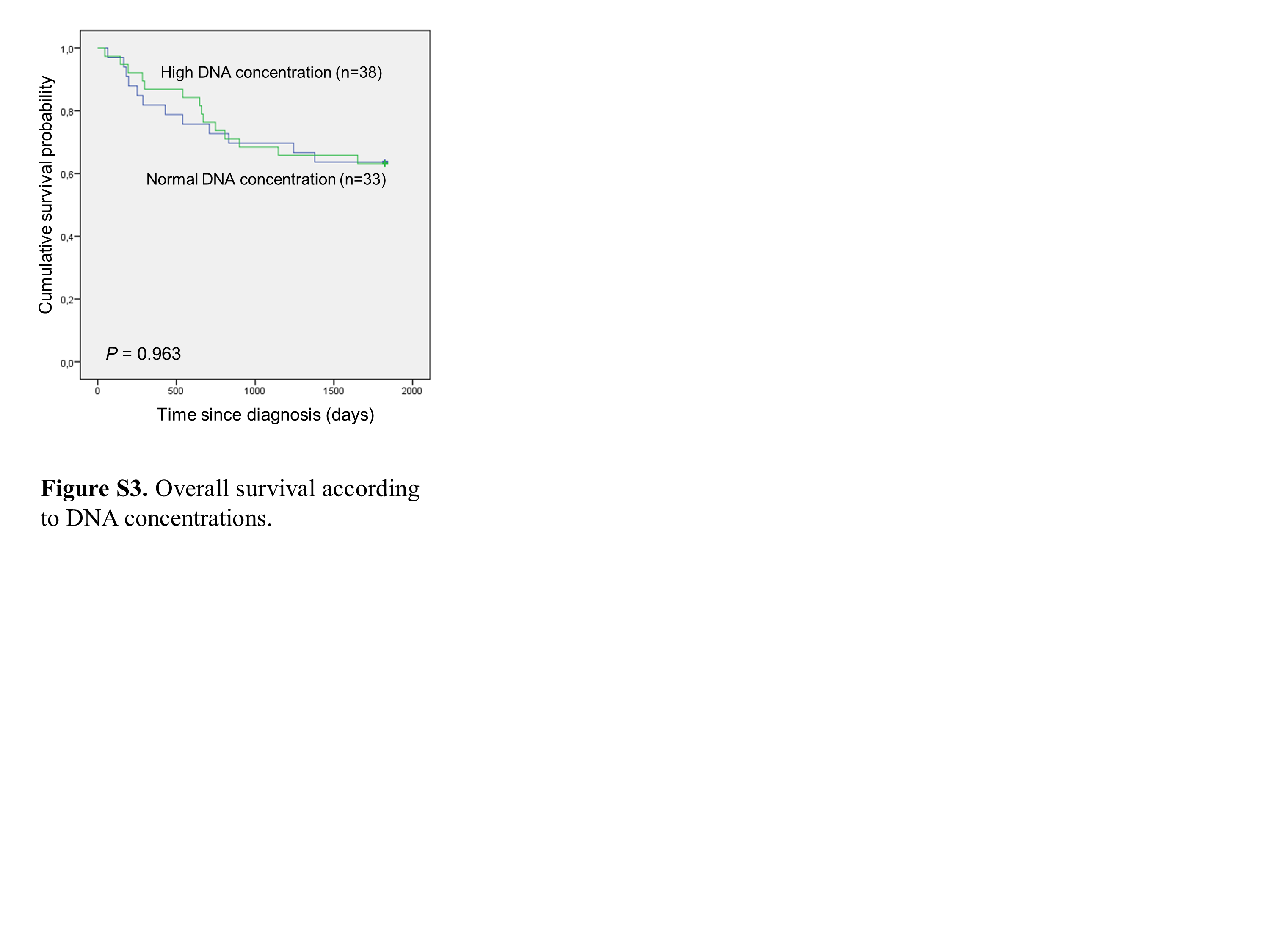

Supplement: Additional file 4: Figure S3. — Overall survival according to DNA concentrations. (TIF 593 kb) [file 13148_2016_261_MOESM4_ESM.tif]

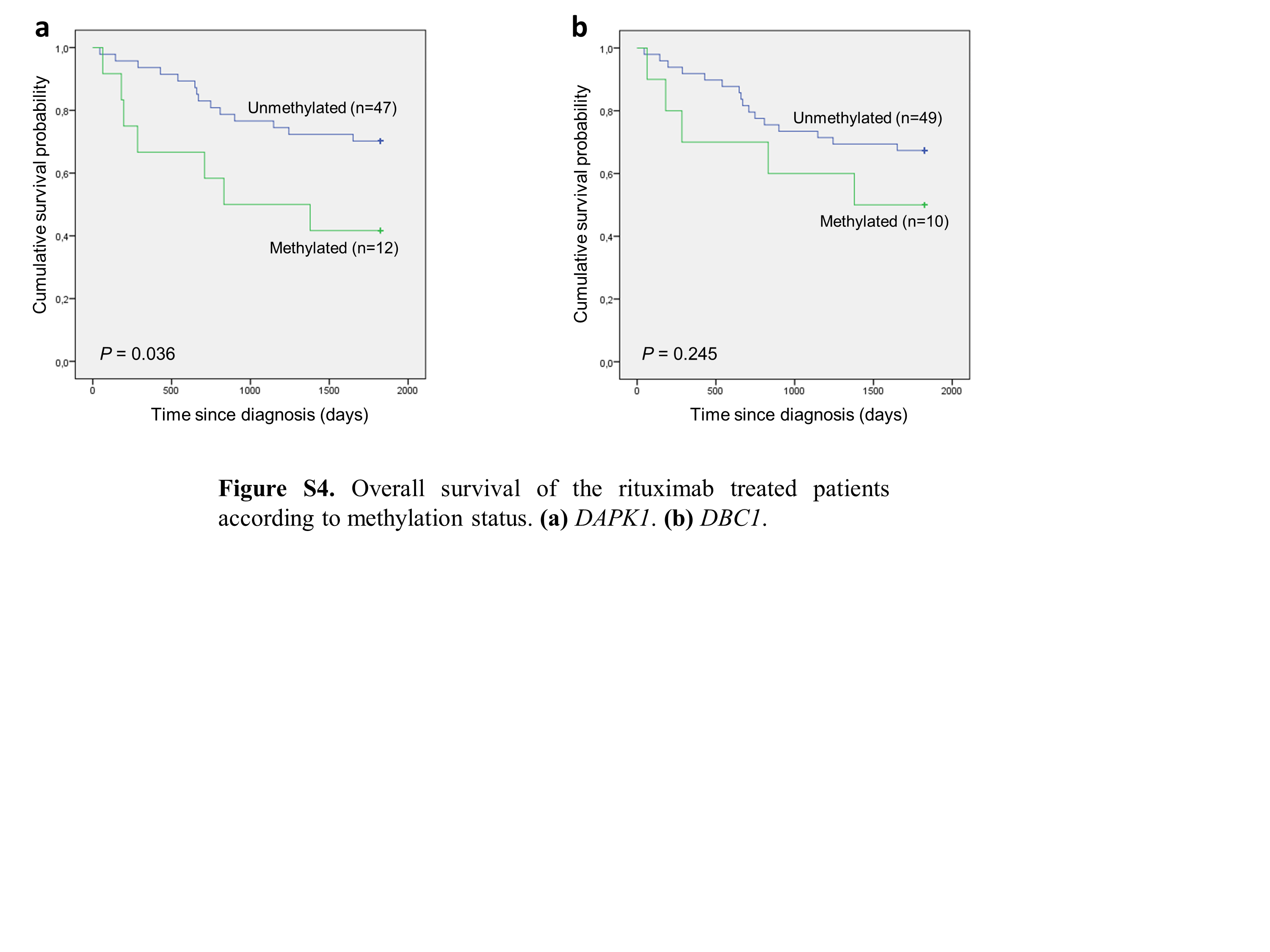

Supplement: Additional file 5: Figure S4. — Overall survival of the rituximab treated patients according to methylation status. (a) DAPKI. (b) DBCI. (TIF 754 kb) [file 13148_2016_261_MOESM5_ESM.tif]
